# Towards a Systems Immunology Approach to Understanding Correlates of Protective Immunity against HCV

**DOI:** 10.3390/v13091871

**Published:** 2021-09-18

**Authors:** Naglaa H. Shoukry

**Affiliations:** 1 Centre de Recherche du Centre Hospitalier de l’Université de Montréal (CRCHUM), Tour Viger, Local R09.414, 900 Rue St-Denis, Montréal, QC H2X 0A9, Canada; naglaa.shoukry@umontreal.ca; 2 Département de Médecine, Faculté de Médecine, Université de Montréal, Montréal, QC H2X 0A9, Canada

**Keywords:** protective immunity, HCV, transcriptomic, systems immunology

## Abstract

Over the past decade, tremendous progress has been made in systems biology-based approaches to studying immunity to viral infections and responses to vaccines. These approaches that integrate multiple facets of the immune response, including transcriptomics, serology and immune functions, are now being applied to understand correlates of protective immunity against hepatitis C virus (HCV) infection and to inform vaccine development. This review focuses on recent progress in understanding immunity to HCV using systems biology, specifically transcriptomic and epigenetic studies. It also examines proposed strategies moving forward towards an integrated systems immunology approach for predicting and evaluating the efficacy of the next generation of HCV vaccines.

## 1. Introduction

Acute hepatitis C virus (HCV) infection resolves spontaneously in approximately 30% (15–45%) of infected individuals, whereas the remaining 70% (55–85%) of persons develop persistent infection and progressive chronic liver disease and are at risk of developing liver cancer [1]. In 2016, the World Health Organization proposed a strategy to eliminate hepatitis as a public health problem by 2030 [2]. Elimination (a reduction to zero new cases in a defined geographical area) relies on the use of highly effective direct-acting antivirals (DAA) that can achieve complete cure in ~95% of treated subjects [3]. However elimination of HCV and potentially its eradication (a complete and permanent worldwide reduction to zero new cases) are unlikely to occur without vaccines that can limit new virus transmission [4], especially in high-risk populations who have reduced access to testing and treatment and who are at higher risk of reinfection [5]. A highly immunogenic T-cell-based vaccine against HCV recently failed to prevent chronic infection in a phase 2 clinical trial in people who inject drugs (PWID) [6]. Therefore, there is an urgent need to dissect the key components of protective immunity against HCV in real-life conditions and to identify elements that were missing in the vaccine-induced immune response.

Systems immunology approaches that employ integrated interdisciplinary methods to define the interactions between the different cellular and molecular components of the immune system have become powerful tools for profiling immune responses to vaccines and viral infections [7,8,9,10,11,12,13,14,15,16,17]. HCV represents an interesting model to apply systems immunology approaches to study immunity against a human viral infection with two dichotomous outcomes, where the correlates of protective immunity in spontaneously resolved versus chronic infection can be examined. Furthermore, chronic HCV infection can be completely cured using DAA, thus offering a unique opportunity to assess the reversal of immune exhaustion/dysfunction post-virus clearance. Finally, neither spontaneous nor DAA-mediated HCV clearance protects against reinfection. This is partly due to the large number of viral variants [18] that are continuously produced during infection [19,20,21] and transmitted among high-risk populations, resulting in mixed infections or superinfections [22]. Indeed, high-risk individuals such as PWID continue to be exposed and reinfected, thus presenting an opportunity to examine the correlates of protective immunity against a highly variable virus in a natural rechallenge experiment. Recently, several studies examined the transcriptomic and epigenetic changes in the context of HCV infection with different outcomes and post-DAA-treatment. Here, I will summarize these studies and propose an integrated systems immunology approach to define correlates of protective immunity against HCV.

## 2. Overview and Comparison of Systems-Transcriptomic Methods

Over the past two decades, different methods have been developed and used to measure gene expression by quantifying mRNA levels in transcriptomic analysis, as well as gene expression modifications through epigenetic analysis. Each method has its own advantages and limitations. The most common methods that were used or are likely to be used in the context of HCV and their limitations are summarized in this section.

### 2.1. Microarrays

Microarrays are the earliest form of high-throughput technology used in transcriptomic analysis. This approach is based on the use of many probes (typically thousands) of specific single-strand DNA fragments, corresponding to genes of interest that are loaded on a chip [23]. These probes can bind fluorescently labeled complementary DNA (cDNA) reverse transcribed from mRNA samples of interest. The intensity of the fluorescence corresponds to the expression level of the corresponding transcript within the mRNA [23]. However, microarrays require a relatively high mRNA input, and they can only detect specific predefined transcripts that are included on the chip.

### 2.2. RNA-Sequencing (RNA-Seq)

The development of next-generation sequencing (NGS) led to its application to RNA-sequencing (RNA-seq), in which the entire transcriptome can be examined. This method is based on the direct sequencing of fragmented cDNA libraries reverse transcribed from the mRNA of the sample of interest. Sequence reads are then mapped to the genome and the data are further transformed and processed to obtain read counts that reflect expression levels within the sample. RNA-seq has completely replaced microarrays due to several key advantages. First, it provides a significantly superior dynamic range for measuring expression across a wide range of transcript levels with less input RNA, making it more suitable for rare patient samples and for the detection of transcripts of low abundance [24]. Second, because it relies on direct sequencing as compared to the use of predefined probes in microarrays, it is suitable for the detection of novel genes or transcripts of interest that may not be represented on the microarray chip. Third, the direct sequencing approach makes RNA-seq highly suitable for use in surrogate animal models of different species such as the equine hepacivirus (EqHV) model [25]. Finally, RNA-seq data provide additional analysis opportunities for important biological information, e.g., the comparison of differential splicing across samples [26,27], functionally relevant single nucleotide polymorphism (SNP) analysis [28] and RNA editing events [29,30,31]. Both microarrays and RNA-seq can be performed on either total mRNA extracted from tissues (e.g., liver tissue), total peripheral blood mononuclear cells (PBMCs) or purified/sorted cells of interest (e.g., total CD8+ T cells or tetramer+ CD8+ T cells). RNA-seq analysis, performed on whole tissue or populations of cells, reflects the averaged gene expression across all cells and is thus termed ‘bulk RNA-seq’. It is useful to detect differences in different experimental conditions, disease outcomes and/or tissues. It is now also possible to infer the relative frequencies of different cellular populations from bulk RNA-seq data in a process known as CIBERSORT [32]. However, the sensitivity of the detection of expression signals associated with a specific cellular subset is dependent upon the frequency of that subset and the levels of expression of the transcript of interest. Hence, signals from rare but key cellular populations may be missed.

### 2.3. Single Cell RNA-Seq (scRNA-Seq)

The advent of scRNA-seq has provided unprecedented opportunities for examining transcripts at the single-cell level and for defining the heterogeneity of cellular populations within a tissue or specific cellular subsets (e.g., HCV-specific CD8+ T cells). This process is based on partitioning individual cells into plates or droplets, where they are lysed and labeled with a unique identifier and then processed using procedures similar to those used for bulk RNA-seq. This method can also be coupled with analysis of the T or B cell receptors, thus providing the capacity to analyze the T or B cell repertoire and to track the expansion of specific T or B cell clones during the course of infection or in different tissue compartments. This technology is highly useful in understanding the interaction of individual cells with their microenvironment and has been successfully used to characterize the heterogeneity of macrophage populations in the liver and their evolution during different stages of fibrosis [33,34,35]. However, the additional processing steps involved in scRNA-seq may differentially affect the viability and consequently the representation of certain cellular populations in the final dataset, leading to interpretation bias [36,37]. In addition, scRNA-seq uses a low cellular and RNA input and this is associated with low RNA capture efficiency and a phenomenon known as “dropout”, occurring when a gene is observed at a moderate or even high expression level in one cell but is not detected in another cell [38]. As a result, scRNA-seq data are of lower resolution and exhibit higher technical noise than bulk RNA-seq [38]. In addition, microarrays, bulk RNA-seq and scRNA-seq are all prone to ‘batch effect’ related to variations induced by non-biological factors during different experimental batches. These issues represent challenges for the computational analysis of transcriptomic data. Several methods are currently available to correct for some of these issues (e.g., batch effect) and new bioinformatic analysis tools are constantly being developed to overcome these limitations, but they all need to be carefully considered and customized in data analysis and interpretation for each experiment. In summary, both bulk and scRNA-seq have their advantages and limitations and the choice of the method to use should carefully consider the research question and the limitations associated with each method.

### 2.4. Combined Transcriptomic and Proteomic Analysis Methods

Transcriptomic data requires validation at the protein level. Advances in high-dimensional flow cytometry and mass cytometry (cytometry by time-of-flight mass spectrometry (CyToF)) and the capacity to run high-resolution panels have provided additional dimensions for data validation but are still limited in terms of the different antibody combinations that can be used (typically 40–50 markers) [39]. Recent methods, which have not yet been applied to HCV, combine transcriptomic and proteomic analysis. These include cellular indexing of transcriptomes and epitopes by sequencing (CITE-Seq) and RNA expression and protein sequencing (REAP-Seq) [40,41]. Both methods combine RNA sequencing with the use of antibodies that are labeled with a specific barcode corresponding to the protein of interest, and a stretch of adenine bases that serve as a starting point for RNA sequencing. The two technologies differ only in the conjugation methods used to link the DNA barcode to the antibodies. They are used to obtain quantitative and qualitative information on surface proteins at the single-cell level, together with their transcriptome. Thus, different cellular populations of interest (e.g., different memory T cell subsets) can be identified and examined within the same experiment. CITE-Seq was shown to be qualitatively and quantitatively similar to flow cytometry [40]. These two methods can allow the detection of more markers as compared to high-resolution flow cytometry or CyToF (~100) but are still limited to the detection of cell surface molecules and are influenced by the quality of the antibodies used to detect the markers of interest [42].

### 2.5. Epigenetic Analysis Methods

Changes in gene expression may be partially due to epigenetic changes that limit chromatin accessibility for gene transcription. One method that was used to assess genome-wide chromatin accessibility in HCV studies is the assay for transposase-accessible chromatin with high-throughput sequencing (ATAC-seq) [43]. This assay is based on the use of hyperactive Tn5 transposase, which preferentially inserts sequencing adapters into open chromatin regions, allowing the direct construction of a sequencing library. Sequencing reads are used to infer regions of increased accessibility and map regions of transcription factor binding and nucleosome position. The number of reads per region correlates with how open the chromatin is at this region. This method uses a low cell input with no need for extensive preparation. It can also be applied at the single-cell level as single-cell ATAC-seq (sc-ATAC-seq). One of the limitations of ATAC-seq is that open chromatin information alone is not sufficient to infer the binding of transcription factors that do not bind to a specific motif or those that bind motifs that can bind multiple transcription factors. In that case, other assays should be used, such as chromatin immunoprecipitation sequencing (ChIP-seq), in which chromatin is crosslinked using formaldehyde, then fragmented and the DNA-protein complexes of interest are immunoprecipitated by antibodies and then sequenced [43].

### 2.6. Spatial Transcriptomics

It is becoming increasingly important to examine cells in the context of their tissue microenvironment to understand their spatial interactions [17,44]. Although all the methods described above provide important information about the transcriptomic and functional signatures of different cells, they do not examine their spatial distribution and interactions within tissues. Recent advances in high-resolution tissue imaging and spatial transcriptomics have made it possible to examine cellular interactions and their single-cell transcriptomic profiles within tissues. Spatial transcriptomics localizes mRNA transcripts to precise spatial locations (single cells or regions) in the tissue of interest [45]. This can be combined with immunohistochemistry imaging techniques and image integration applications to visualize the interaction of the immune cells within the tissue of interest [46]. The potential applications of these methods in studying the liver are reviewed elsewhere [35]. Although there is an increasing shift away from diagnostic liver biopsies in favor of non-invasive testing, spatial transcriptomics can be applied to archived human and chimpanzee liver tissue samples from prior studies and clinical trials. Furthermore, these methods can also be used in surrogate animal models such as the rat hepacivirus model that recapitulates the dynamics of HCV infection to a great extent and would be a valuable model to examine immune responses and their spatial interactions within the liver microenvironment [47,48].

## 3. Systems Biology Studies of the Immune Response during Acute and Chronic HCV Infection

### 3.1. Studies of Liver Tissue and Total PBMCs

Multiple studies in humans and chimpanzees have used systems biology approaches to characterize the immune response during acute HCV infection progressing to either spontaneous resolution or persistent infection. Early microarray studies in the chimpanzee model observed the rapid induction of intrahepatic interferon signals, as demonstrated by an increase in the expression of interferon-stimulated genes (ISGs) in the liver shortly after experimental infection, irrespective of the outcome [49,50]. Transient and sustained viral clearance was associated with the induction of IFN-γ-induced genes, as well as genes involved in antigen processing and presentation and the adaptive immune response [49]. These signatures correlated with the strength of the intrahepatic immune response in these animals [51]. Similarly, in cross-sectional human studies, IFN-γ-induced genes, ISGs, genes for chemokines and their receptors, as well as genes associated with cellular immunity, were upregulated in liver biopsies obtained during acute HCV infection as compared to healthy liver tissue [52]. Longitudinal RNA-seq analysis of PBMCs from acutely infected PWID revealed prominent type I interferon (ISG) and inflammatory signatures [53]. Innate immune gene expression rapidly returned to pre-infection levels upon spontaneous viral clearance but remained elevated in those who developed persistent viremia [53]. Comparative analyses demonstrated common signatures with those of flaviviruses yellow fever and dengue [53]. Recent RNA-seq analysis of the liver and peripheral blood of horses infected with equine hepacivirus (EqHV), which is highly related to HCV, demonstrated an ISG signature in the liver [25]. However, only minimal perturbations in adaptive immune cell signatures were detectable in the peripheral blood despite the fact that EqHV-specific T cells were identified following specific peptide stimulation [25].

Studies performed on liver biopsies and/or PBMCs obtained during chronic HCV infection demonstrated that ISGs remain upregulated but that their level was variable among different patients [52,54,55,56]. Furthermore, a set of ISGs were identified that correlated with the reduced response to IFN-based therapies. Specifically, USP18 (a negative regulator of IFN-α signaling), was upregulated in liver samples of chronic HCV patients who were unresponsive to IFN therapy [52,54]. ISG levels correlated with specific alleles of single nucleotide polymorphism in the IL-28B/IFNλ3 locus encoding the type III interferon IFNλ4 [57,58] (for a detailed review see [59]).

These studies performed on whole liver tissue or unfractionated PBMCs provided snapshots of the overall immune response during acute HCV infection and demonstrated that innate immune responses are induced early, irrespective of infection outcome, and remain upregulated during chronic infection. However, signals associated with adaptive immunity, although detectable in some studies, were diluted and it was not possible to identify signatures that can predict infectious outcomes, suggesting that protective immune functions are limited to a small cellular subset.

### 3.2. Studies of CD8+ T Cells during Acute HCV Infection

T cell responses during acute HCV infection have been extensively studied and are reviewed elsewhere [60,61,62]. The consensus from these studies is that the T cell response is generally delayed, appearing in the peripheral blood around 6–8 weeks post-infection and may be detectable a week or two earlier in the liver. A broad CD4+ and CD8+ T cell response targeting multiple epitopes and that is polyfunctional (producing multiple cytokines and effector functions) is essential for the spontaneous clearance of acute HCV (reviewed in [62]). HCV resolvers develop long-lived virus-specific CD8+ memory T cells expressing the memory marker CD127 [63,64,65,66], and HCV-specific CD4+ T cells producing IL-21 were associated with spontaneous clearance and enhanced survival of HCV-specific CD8+ T cells [67].

The immune response during the early phase of acute infections that become chronic is not substantially different, as relatively broad HCV-specific CD4+ and CD8+ T cell responses are generated early and may contribute to the transient control of viremia, but this response is short-lived. Specifically, HCV persistence is associated with the sudden failure and disappearance of HCV-specific CD4+ helper T cell responses. The lack of CD4+ T cell help compromises CD8+ T cell function(s) and facilitates the emergence of viral escape mutants in targeted CD8+ T cell epitopes [68,69,70] resulting in the establishment of persistent viremia. CD8+ T cells that recognize intact epitopes that have not mutated become exhausted and upregulate T cell activation and exhaustion markers such as programmed death-1 (PD-1), T-cell immunoglobulin and mucin-domain containing-3 (TIM-3), cytotoxic T-lymphocyte protein 4 (CTLA-4), 2B4, CD160, KLRG1, T-cell immunoreceptor with Ig and ITIM domains (TIGIT) and CD39 [62,71]. They also upregulate the nuclear factors eomesodermin (EOMES) and thymocyte selection-associated HMG box (TOX), downregulate T-bet, and express variable levels of the T cell factor 1 (TCF1) that delineates different CD8+ T cell subsets (discussed below) [72,73,74,75]. The persistent antigenic stimulation and exhaustion lead to the gradual loss of proliferative capacity, cytokine production and cytotoxicity. In contrast, CD8+ T cells targeting epitopes that have mutated are no longer exposed to persistent antigenic stimulation and exhibit a CD127+ memory T cell phenotype, similar to that of memory T cells generated following spontaneous resolution [76]. As chronic infection is established, the frequencies of HCV-specific CD8+ and CD4+ T cells decline in peripheral blood and are more enriched in the liver [77,78,79,80].

To decipher the mechanisms underlying T cell failure during acute HCV infection and to identify predictors of the infectious outcomes, Woloski et al. [81] performed a microarray transcriptomic study of HCV-specific CD8+ T cells sorted from the peripheral blood using MHC class I tetramers during early and late acute HCV infections progressing to spontaneous resolution, chronic infection with intact epitopes, or chronic infection with escaped/mutated epitopes. They observed dysregulation of metabolic processes early in infections progressing to chronicity. This dysregulation was linked to changes in the expression of genes related to nucleosomal regulation of transcription, T cell differentiation and inflammation. This signature also correlated with age, sex and the presence of HCV-specific CD4+ T cells in the infected subjects. Importantly, samples from individuals with escaped epitopes only partially shared this metabolic and immune dysfunction signature [81]. This metabolic dysfunction signature was also partly shared by exhausted CD8+ T cells in the lymphocytic choriomeningitis virus (LCMV) mouse infection model, suggesting a common mechanism [81,82]. Another study by Barili et al. [83] using a similar strategy also demonstrated that in early acute HCV infection, exhaustion-committed virus-specific CD8+ T cells display the upregulation of transcription signatures associated with impaired glycolytic and mitochondrial functions. After the establishment of chronic infection, HCV-specific CD8+ T cells were characterized by broad downregulation of genes associated with wide metabolic and anti-viral function impairment [83].

### 3.3. Studies of CD8+ T Cells during Chronic HCV Infection and DAA Therapy

CD8+ T cell exhaustion during chronic HCV is not an all-or-none phenomenon but rather a spectrum of different degrees of impairment of antiviral functions associated with the expression of different levels of T cell exhaustion and memory markers [67,84]. TCF1^+^CD127^+^PD1^+^T-bet^lo^ HCV-specific CD8+ T cells expressing both exhaustion and memory markers were described in chronically infected subjects and termed ‘memory-like’ or ‘stem-like’ T cells because of their ability to retain proliferative capacity [85,86]. DAA-mediated HCV clearance was associated with the preferential maintenance of memory-like cells [85]. This is consistent with the fact that DAA treatment results in only partial reversal of T cell exhaustion, whereby proliferative responses are restored but the capacity to produce cytokines or cytotoxicity are not [85,87,88].

To understand the molecular mechanisms and pathways underlying CD8+ T cell dysfunction during chronic HCV and whether these pathways are reconstituted following DAA therapy and clearance, a series of recent elegant studies examined this question in different cohorts using multiple systems biology approaches [89,90,91]. These studies examined the transcriptomic, phenotypic and functional signatures of MHC class I tetramer-sorted HCV-specific CD8+ T cells from subjects with chronic HCV infection pre- and post-DAA-treatment. As a control of another acute resolving infection, some also examined influenza-specific CD8+ T cells in the same individuals [90,91] and HCV-specific CD8+ T cells from spontaneous resolvers [89,90,91]. Using bulk RNA-seq and high-resolution flow cytometry analysis, three different HCV-specific CD8+ T cell subsets could be identified in subjects with chronic HCV: CD127^hi^, CD127^int^ and CD127^lo^ [89]. CD127^hi^ cells and CD127^lo^ cells had the molecular and phenotypic signatures of memory-like and exhausted CD8+ T cells, respectively [89]. These memory-like and exhausted signatures were shared with CD8+ T cells in the LCMV mouse model and cancer [89,90,92]. scRNA-seq together with T cell receptor typing identified a progenitor–progeny relationship between CD127^hi^ memory-like and CD127^lo^ exhausted CD8+ T cells via a CD127^int^ intermediate stage [89].

HCV-specific CD8+ T cells targeting escaped epitopes were also examined. Given that immune escape occurs early in most cases, the premise was that CD8+ T cells targeting escaped epitopes would have been subjected to a shorter period of antigenic stimulation and exhaustion. This approach would also allow the distinction between antigen-mediated exhaustion versus exhaustion mediated by the inflammatory environment due to ongoing HCV infection and replication. Indeed, the core exhaustion signature identified in exhausted CD8+ T cells was less pronounced in CD8+ T cells targeting escaped epitopes, suggesting that this signature was antigen-dependent [89,90]. By examining samples before and after DAA-mediated HCV cure, the authors observed the downregulation of some exhaustion markers such as PD-1 and the preferential maintenance of memory-like virus-specific CD8+ T cells [89,90], as previously described [85]. These memory-like T cells harbored the same transcriptomic signatures before and after cure, suggesting a permanent “exhaustion scar” that cannot be reversed by HCV cure [89,90]. This exhaustion scar was maintained for up to 3 years post-DAA-mediated HCV clearance, in a subset of patients [90].

In a companion study, Yates et al. used ATAC-seq to examine the epigenetic state and chromatin-accessible regions (ChARs) in the same type of samples [91]. They identified a core regulatory program in exhausted HCV-specific CD8+ T cells. This epigenetic program was conserved across other chronic viral infections such as human immunodeficiency virus (HIV) and LCMV [91,92]. Furthermore, this program was largely irreversible for up to 80 weeks following DAA-mediated clearance, suggesting that exhausted HCV-specific T cells were locked into a permanent dysfunctional state [91]. By further examining ChARs pre- and post-DAA-treatment, they were stratified into three patterns, scarred, reversed or gained, that regulate functionally distinct class of genes. Scarred regions were enriched for genes associated with T cell exhaustion such as HIF1α and NFAT, reversed regions were enriched for genes and pathways as associated with T cell receptor and PD-1 signaling, and gained regions were enriched for genes associated with memory T cell development such as IL-7 and IL-2 signaling [91]. To further understand this process, they developed an algorithm to infer super-enhancer activity directly from chromatin accessibility and showed that epigenetic scars include super-enhancer elements near exhaustion-associated transcription factors such as TOX and HIF-1α, which are known to be critical to maintaining the state of exhaustion [91]. Finally, they observed partial epigenetic remodeling within HCV-specific CD8+ T cells targeting escaped epitopes [91], again confirming the antigen-dependent nature of T cell exhaustion.

These results suggest that CD8+ T cells that are maintained post-DAA-mediated HCV clearance will not be protective upon reinfection and may not respond to vaccination. Interestingly, one study in a chimpanzee treated with DAA then experimentally rechallenged demonstrated that pre-existing intrahepatic HCV-specific CD8+ T cells were narrowly focused and failed to prevent viral persistence [93]. Both the transcriptomic and epigenetic profiles observed in HCV-infected subjects were recapitulated in another study in the LCMV model, thus underscoring common exhaustion molecular mechanisms in different infection models [92]. Experiments in the LCMV mouse model demonstrated that recovered exhausted CD8+ T cells adoptively transferred into naïve mice and rechallenged with the virus possessed inferior recall capacity as compared to conventional memory T cells, which is only partially improved with PD-L1 blockade [92]. New studies examining the immune response and reversal of exhaustion following reinfection in DAA-treated subjects will be essential to validate these findings in human HCV infections.

**Figure 1:**
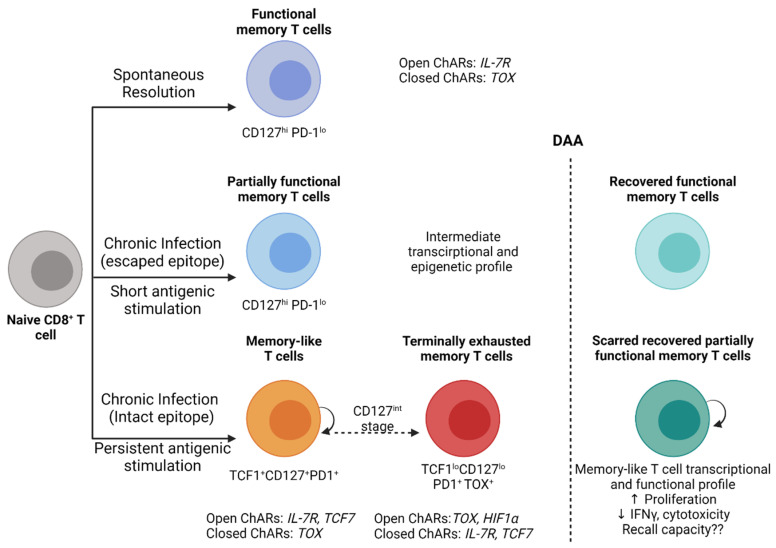
Model of CD8+ T cell exhaustion and functional restoration during HCV infection and DAA therapy. Following the spontaneous clearance of acute HCV infection, functional memory CD8+ T cells (CD127^+^PD-1^lo^) develop and maintain epigenetic signatures with open chromatin accessibility regions (ChARs) that facilitate rapid recall responses. In individuals who develop chronic infection, with CD8+ T cells targeting escaped epitopes and exposed to a short period of antigenic stimulation recover an intermediate phenotype, transcription and an epigenetic profile that resembles to a great extent functional memory T cells. CD8+ T cells targeting intact epitopes remain persistently stimulated by antigens and develop into memory-like T cells (TCF1^+^CD127^+^PD1^+^) that retain their proliferative capacity and develop into terminally exhausted memory T cells (TCF1^lo^CD127^lo^PD1^+^TOX^+^) through a CD127^int^ stage. These exhausted CD8+ T cells exhibit transcriptional and epigenetic signatures similar to exhaustion profiles reported in HIV, LCMV and cancer. DAA-mediated virus clearance and the removal of persistent antigenic stimulation reverses most of the functional and epigenetic changes in CD8+ T cells targeting escaped epitopes. In contrast, CD8+ T cells targeting intact epitopes maintain an exhaustion scar and only partially recover antiviral functions with restored proliferation but not cytokine production or cytotoxicity. These exhaustion scars are long-lived for up to 3 years post-virus clearance. The recall capacity of recovered T cells upon HCV reinfection remains unknown but data from the LCMV model suggest that it will be compromised. Created with BioRender.com.

Altogether, these studies clearly demonstrate the development of a permanent/locked antigen-dependent exhaustion scar in HCV-specific CD8+ T cells with the establishment of chronic infection. This scar impairs the antiviral capacity of virus-specific CD8+ T cells long after virus clearance. The reversibility of this scar is likely dependant upon the period of antigen exposure, and early antigen removal (as in the case of escaped epitopes) may facilitate the unlocking and reversal of this exhaustion scar and functional restoration (Figure 1). Further studies evaluating the protective capacity of natural versus treatment-induced memory T cells during real life exposure and reinfection post-DAA treatment are warranted.

### 3.4. Studies of CD4+ T Cells during HCV Infection and DAA Therapy

Mechanisms underlying the dysfunction of HCV-specific CD4+ T cells and their failure to help CD8+ T cells are understudied, mainly due to the difficulty in detecting antigen-specific CD4+ T cells. Studies using a limited number of MHC class II tetramers demonstrated that HCV-specific CD4+ T cells express a narrower range of T cell inhibitory receptors compared with CD8+ T cells, with high expression levels of PD-1 and CTLA-4 [94]. HCV-specific CD4+ T cells were also phenotypically and functionally similar during early resolving and persistent infection in individuals with different outcomes, suggesting that these two receptors may be markers of activation rather than exhaustion at this early stage [94,95]. Following spontaneous clearance, virus-specific CD4+ T cells downregulated PD-1 and CTLA-4 and developed into memory T cells. In contrast, HCV persistence led to the sustained upregulation of PD-1 and CTLA-4 and the eventual disappearance of HCV-specific CD4+ T cells from the peripheral blood [94,95]. A subset of HCV-specific CD4+ T cells in the peripheral blood also express markers of follicular helper T cells (Tfh), a population with an important role in helping B cells and in antibody production [96,97]. These circulating Tfh (cTfh) cells produce IL-21 during acute infection and, although they disappear from the circulation with HCV persistence, they remain detectable in the liver and maintain the Tfh phenotype [98]. RNA-seq analysis on three subjects before, during and after DAA therapy observed the convergence of the transcriptional profile of HCV-specific CD4+ T cells towards that of cTfh, which is consistent with phenotypic analysis demonstrating that HCV-specific CD4+ T cells with a Tfh phenotype are preferentially maintained following DAA-mediated HCV clearance [99]. Additional studies examining the HCV-specific CD4+ T cells and novel methods to identify and characterize in detail this important cell subset relative to the kinetics of CD8+ T cell exhaustion and dysfunction are needed to understand the role of CD4+ T cell help in mounting and sustaining an efficient immune response against HCV.

### 3.5. Studies of B Cells

Early studies of the antibody response against HCV suggested that anti-HCV antibodies are delayed, short-lived in resolvers and do not always correlate with spontaneous resolution [100,101]. Advances in the development of HCV pseudoparticles (HCVpp), HCV entry factor transgenic mice, the isolation of broadly neutralizing antibodies (nAbs) and the elucidation of the HCV E2 crystal structure have allowed better appreciation and understanding of the important role of nAbs during acute and chronic HCV infection, as well as reinfection, and are reviewed elsewhere [102,103,104]. However, little is known about HCV-specific B cells and their evolution during HCV infections with different outcomes. The recent development of E2-tetramers has allowed the visualization and analysis of this important cellular population during acute infection [105,106]. E2-specific memory B cells (MBCs) from spontaneous resolvers peaked early after infection (4–6 months), following the expansion of activated cTfh expressing interleukin 21. This was associated with the transient development of nAbs against HCV [106]. In contrast, E2-specific MBCs from chronically infected subjects expanded later, following the establishment of persistent infection (>1 year), in the absence of significantly activated cTfh expansion [106]. scRNA-seq performed on E2-specific MBCs from resolvers and chronically infected subjects sorted at their respective peaks of expansion demonstrated similar patterns of gene expression [106]. These data suggested that CD4+ T cell help plays an important role in the early expansion of HCV-specific MBCs and the production of nAbs that may contribute to spontaneous resolution. Additional studies examining in detail the transcriptional and functional profiles of HCV-specific MBCs and cTfh are required. Furthermore, the ability to biopsy and examine lymph nodes using fine-needle aspirates would provide key insights on the Tfh–B cell interaction in the germinal centers.

## 4. Proposed Integrated Systems Immunology Approach to Define Correlates of Protective Immunity against HCV and Response to Vaccination

**Figure 2:**
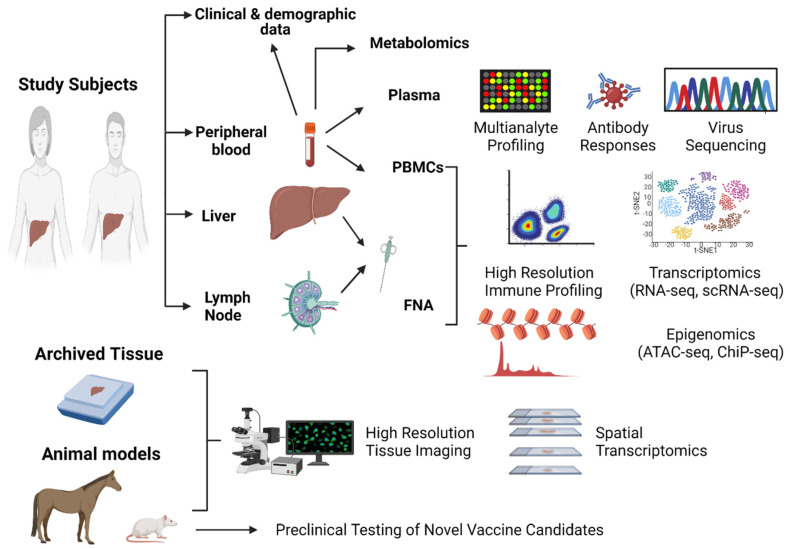
Proposed integrative strategy for a systems immunology approach to future HCV immunological studies and vaccine clinical trials. Blood samples, liver and lymph node fine-needle aspirates (FNA), archived liver tissue samples and animal models can be used in an integrative approach to identify correlates of protective immunity and inform vaccine development. Created with BioRender.com.

As reviewed in the previous sections, there has been tremendous progress in the use of systems transcriptomic approaches to understand protective immunity against HCV. The data collected so far have revealed mechanisms of CD8+ T cell functionality and exhaustion that are shared with other viral infections and cancer. Such studies can inform and be informed by studies in other models. They are also crucial to establishing benchmark signatures and parameters that should be achieved by the next generation of vaccines. However, most studies are still performed independently, and an integrative approach should be applied to future cohort studies and vaccine clinical trials through a systematic and standardized process in order to enhance the data that can be extracted and their comparisons among different studies. This can be guided by recent collaborative studies and approaches applied to understanding immunity against SARS-CoV2, the development of predictors of disease outcomes and the evaluation of the efficacy and durability of vaccine-induced responses [17,107,108]. The existence of multiple cohorts and sample repositories offers a unique opportunity for large collaborative studies in which results can be validated across different cohorts. In this section, I outline a proposed approach to guide future cohort studies and vaccine clinical trials (Figure 2). The different types of key biological samples/models that can be used and analyses that can be performed are discussed below.

### 4.1. Peripheral Blood

Blood samples are the easiest to collect and can be used directly to obtain clinical data and to examine the metabolome. However, the amount of blood that can be drawn at a regular visit is limited and the ability to perform leukapheresis at key timepoints during infection and vaccine clinical trials should be considered. Peripheral blood can be processed to separate plasma and PBMCs. Plasma can be used for multianalyte testing of inflammatory mediators and serum markers, antibody profiling via both ELISA and neutralization assays using standardized protocols, reagents and HCVpp panels. Plasma can also be used to perform virus sequencing using full-length NGS approaches to understand the interaction between different viral variants and the adaptive immune response [109]. Indeed, the high variability of HCV remains a major hurdle for vaccine development. As demonstrated by transcriptomic studies, escape mutations in targeted epitopes lead to an intermediate transcriptomic signature, bearing features of both spontaneous resolution and exhaustion. Furthermore, full-length sequencing can be used to track transmission clusters in high-risk groups before and after vaccination.

Total PBMCs can be used to monitor the overall immune response using standardized assays and peptide sets, as well as MHC class I and II tetramers. HCV-specific T cells can be identified and sorted using MHC class I and II tetramers or activation surface markers. Similarly, HCV-specific B cells can be identified and sorted using E2 tetramers. These sorted cells can be used to perform bulk RNA-seq to understand the overall transcriptomic signature in these different populations and scRNA-seq combined with B cell or T cell repertoire analysis to monitor the evolution of different T or B cell clones during various stages of infection. Sorted B cells can also be used to clone and express HCV-specific antibodies and assess their neutralization profile using standardized HCVpp panels. Systems serology approaches and algorithms can be applied using various assays (ELISA, HCVpp neutralization, etc.) to assess the B cell response and to evaluate the efficacy of next-generation vaccines, as has been proposed for HIV [110].

### 4.2. Liver and Lymph Nodes

The peripheral blood only reflects part of the immune response. As discussed above, it has become increasingly important to study the immune cell interactions in tissues of interest, whether it is the liver as the site of infection or the lymph nodes, where key interactions in the germinal centers drive the antibody response. To examine the immune response in the liver, it is still possible to obtain fine-needle aspirates during clinical trials and compare the HCV-specific T and B cells in the blood and liver compartments at the single-cell level. Fine-needle aspirates of draining lymph nodes are also well integrated in clinical trials for various vaccines and would provide key information about the interaction between CD4 helper T cells and B cells. Archived liver biopsy samples can be used for spatial transcriptomics and tissue imaging in order to understand the interactions of HCV-specific T cells within the liver microenvironment.

### 4.3. Animal Models

Animal models represent a valuable model for the preclinical testing of new vaccines. They also represent a unique complementary tool, in which both systems and classical immunological approaches can be employed to understand the intrahepatic immune response and changes in the liver microenvironment during and post-infection and to assess factors associated with the continued risk of developing liver fibrosis and cancer.

### 4.4. Key Steps and Challenges Moving Forward

Moving forward towards an integrated systems immunology approach for future HCV immunological studies and vaccine clinical trials, there are several key steps to consider, each with its own challenges. First, a team-based approach is essential for the standardization of protocols for sample collection and processing to reduce batch effects and to ensure the reproducibility of results generated in different cohorts. Second, access to sufficient samples for analysis by systematically integrating leukapheresis at key immunological time points is needed. Third, there is a need to build the bioinformatic capacity to filter the huge amount of data that will be generated with the understanding of both the biological processes and the bioinformatic algorithms and to develop novel analysis algorithms that integrate the different types of data into predictive models. Furthermore, as described above, many limitations exist for high-throughput analysis and customized bioinformatics analysis approaches are often needed. Fourth, the validation of the biological mechanisms inferred from the bioinformatics data is critical, using the proper analysis tools and using novel cutting-edge technologies such as high-resolution flow cytometry and tissue imaging with validated sets of antibodies and bioinformatic support for analysis. Finally, the biggest challenge is securing continued funding for the ongoing and new cohorts, as well as vaccine clinical trials and support for the bioinformatic and immunological infrastructures required.

## 5. Concluding Remarks and Future Directions

Systems transcriptomic studies have unravelled important facets of the immune response against HCV. Specifically, innate immune responses are induced early and are maintained during chronic infection. Recent analysis of HCV-specific CD8+ T cells demonstrated the existence of a relatively permanent exhaustion scar in HCV-specific CD8+ cells that is antigen-dependent and that may not be easy to reverse following DAA treatment and thus represents an obstacle to the generation of long-term protective immunity. The role of CD4+ helper T cells and B cells in immunity against HCV and the underlying molecular mechanisms remain virtually unknown. New studies will need to focus on understanding these two subsets, their interaction with CD8+ T cells and their exact contribution to HCV clearance during primary infection and reinfection. A collaborative and integrated systems immunology approach to new cohort studies and vaccine clinical trials is urgently needed. The knowledge gained and infrastructures established to examine immunity against SARS-CoV2 [17,107,108,111] and the responses to vaccination can, with the proper funding and political will, be applied to other viral infections, including HCV.
